# Effectiveness of Hip Muscle Strengthening Compared with Knee Strengthening or No Intervention in Individuals with Patellofemoral Pain Syndrome: A Systematic Review

**DOI:** 10.3390/muscles5030066

**Published:** 2026-09-17

**Authors:** Carlos Contreras-Mora, Sara Cerrolaza-Tudanca, María Soledad Amor-Salamanca, Pedro Antonio Mendoza-Morón, Lara Sanchez-Suarez, María Corral-González, Arturo Cano-Uceda, Nuria Feas-Diaz, José Luis Maté-Muñoz, Pablo García-Fernández, Manuel Rozalén-Bustín

**Affiliations:** 1 Faculty of Nursing, Physiotherapy and Podiatry, Universidad Complutense de Madrid, 28040 Madrid, Spain; 2 Faculty of Health Sciences, Universidad Alfonso X El Sabio, 28691 Madrid, Spainmbustroz@uax.es (M.R.-B.)

**Keywords:** patellofemoral pain syndrome, exercise therapy, hip strengthening, knee strengthening, muscle strength, pain, physical function

## Abstract

**Background**: Patellofemoral pain syndrome (PFPS) is a common musculoskeletal condition in physically active individuals. Although quadriceps strengthening has traditionally been the cornerstone of conservative management, hip-strengthening exercises have gained increasing attention because of their potential to improve lower-limb biomechanics. However, the optimal exercise prescription for PFPS remains uncertain. **Objective**: To evaluate the effectiveness of isolated or combined hip muscle strengthening compared with knee strengthening or no intervention on pain, physical function, and muscle strength in individuals with PFPS. **Methods**: A systematic review was conducted in accordance with PRISMA guidelines and registered in PROSPERO (CRD420261342475). PubMed, PEDro, the Cochrane Library, CINAHL, and Web of Science were searched from inception to 31 December 2025. Randomised controlled trials comparing hip-strengthening interventions with knee strengthening or no intervention were included. Methodological quality, risk of bias, and certainty of evidence were assessed using the PEDro scale, the Cochrane RoB 2 tool, and the GRADE approach, respectively. **Results**: Fifteen randomised controlled trials involving 1002 participants were included. Exercise therapy consistently improved pain, physical function and muscle strength. Both hip- and knee-strengthening programmes were superior to no intervention. Programmes incorporating hip strengthening may provide greater reductions in pain and larger improvements in proximal muscle strength than knee-focused programmes, whereas functional outcomes were generally comparable. Combined hip- and knee-strengthening programmes appeared to provide the most consistent overall clinical benefits. The overall certainty of the evidence was moderate for all outcomes. **Conclusions**: Current evidence supports exercise-based rehabilitation for PFPS, while evidence remains insufficient to determine whether any specific strengthening strategy provides a clear overall advantage. Further high-quality randomised controlled trials are needed to determine the optimal exercise prescription.

## 1. Introduction

Patellofemoral pain syndrome (PFPS) is one of the most common knee musculoskeletal disorders. It is characterised by retropatellar or peripatellar pain that is exacerbated during functional activities that increase the load on the patellofemoral joint, such as running, climbing or descending stairs, performing squats, or sitting for prolonged periods [1,2,3]. Patellofemoral pain is common in several populations, including adolescents and physically active individuals. Among adolescents, reported prevalence ranges from 7% to 28%, while an incidence of approximately 9.2% has also been reported [2]. A systematic review and meta-analysis estimated a pooled point prevalence of 7.2% (95% CI: 6.2–8.3%) among adolescents and 22.7% (95% CI: 17.4–28.0%) among female adolescent athletes [3]. In female athletes, a point prevalence of 16.7% has also been reported in a study of 418 Iranian athletes aged 15–35 years [4]. Randomised trials have also specifically investigated PFPS in physically active young women [5]. Sex-related differences have been reported in specific populations; for example, in military personnel, annual incidence was 6.5% in women and 3.8% in men, while prevalence was 15% and 12%, respectively [2]. Although PFPS was traditionally considered a self-limiting condition, current evidence indicates that a substantial proportion of patients continue to experience symptoms for years. Persistent symptoms may negatively affect physical function, participation in sport, and quality of life, and may increase the risk of patellofemoral osteoarthritis [6,7].

PFPS is now regarded as a clinical condition of multifactorial origin in which biomechanical, neuromuscular and individual factors interact [2]. Among the most widely accepted pathophysiological mechanisms is increased patellofemoral stress secondary to alterations in lower limb mechanics [8,9,10,11,12]. Excessive internal rotation of the femur, increased hip adduction and increased dynamic knee valgus can reduce the patellofemoral contact area and increase joint loads, thereby promoting the onset and persistence of pain [13,14,15]. These alterations have been consistently linked to deficits in strength and neuromuscular control of the hip abductor, extensor and external rotator muscles [15,16,17], which supports the role of proximal structures in regulating the loads transmitted to the patellofemoral joint.

Various observational studies have described kinematic alterations of the lower limb in patients with PFPS, including increased hip adduction and internal rotation, as well as changes in trunk and foot mechanics during functional activities [18,19,20]. Although there is still no consensus on whether these alterations constitute predisposing factors or compensatory mechanisms in response to pain [19,21], there is growing agreement that adequate proximal neuromuscular control is essential for maintaining efficient biomechanics and reducing patellofemoral stress [13,14,15,16,17]. Furthermore, reductions of between 14% and 36% in the strength of the hip abductors and external rotators have been reported in women with PFPS compared with healthy subjects [22,23], supporting the hypothesis that proximal muscle weakness may contribute to the persistence of symptoms. As a result of this evolving understanding of the condition, conservative treatment has also undergone significant changes. Historically, rehabilitation for PFPS has been based on quadriceps strengthening, which remains one of the therapeutic strategies with the strongest scientific support and the fundamental pillar of exercise-based treatment [1,2,15]. However, over the last decade there has been growing interest in strengthening of the hip musculature, particularly the hip abductors, external rotators, and extensors, based on the hypothesis that improved proximal neuromuscular control may influence lower-limb movement patterns and clinical outcomes [13,14,15,16,17]. In this context, several randomised clinical trials have reported clinically relevant improvements in pain and function following hip muscle strengthening programmes, both on their own and in combination with knee-targeted exercises [1,15,16,24].

However, the available evidence has significant limitations. There is considerable heterogeneity between studies regarding the target muscles, intensity, volume, duration of the intervention, progression criteria and level of supervision, which makes it difficult to compare studies and apply the results to clinical practice [1,15,16,24]. Most studies have focused on pain and patient-reported function, whilst the muscle strength, one of the main modifiable factors related to lower limb biomechanics [13,16,17,22,25], has received less attention. Although the available evidence supports the efficacy of therapeutic exercise in PFPS, uncertainties remain regarding which strengthening strategy is the most effective. Previous reviews have included highly heterogeneous exercise programmes or multimodal interventions, making it difficult to determine the specific contribution of hip-muscle strengthening, both in isolation and in combination with exercises targeting the knee. The recent publication of additional randomised controlled trial evidence, including a recent study by Yue et al. [24], warrants an update of the evidence and allows for a more precise assessment of the relative effectiveness of different strengthening strategies.

Consequently, uncertainty remains as to which strengthening strategy provides the greatest benefits in terms of pain, physical function and muscle strength in people with patellofemoral pain syndrome. Resolving this issue is of particular clinical interest, as it may help to optimise the prescription of therapeutic exercise and facilitate decision-making based on the best available scientific evidence.

Therefore, the aim of this systematic review was to evaluate the effectiveness of hip muscle strengthening, either in isolation or in combination, compared with knee muscle strengthening or no intervention, on pain, physical function and muscle strength in patients with patellofemoral pain syndrome. The results of this review aim to provide information to facilitate the selection of evidence-based exercise programmes for the conservative treatment of this condition.

## 2. Methodology

### 2.1. Search Strategy

This systematic review was conducted in accordance with the Preferred Reporting Items for Systematic Reviews and Meta-Analyses (PRISMA) statement [26] and the recommendations of the Cochrane Handbook for Systematic Reviews of Interventions [27]. The completed PRISMA 2020 checklist [28] is provided as Supplementary Material (Table S1). The review protocol was developed a priori and prospectively registered in the International Prospective Register of Systematic Reviews (PROSPERO) under registration number CRD420261342475.

A systematic literature search was carried out between 15 January and 15 February 2026 in five electronic databases: PubMed, the Cochrane Library, Web of Science, the Physiotherapy Evidence Database (PEDro) and the Cumulative Index to Nursing and Allied Health Literature (CINAHL). The search included studies published from the inception of each database up to 31 December 2025.

The search strategy was developed in accordance with the Population, Intervention, Comparison and Outcome (PICO) framework, including participants with patellofemoral pain syndrome (P), hip muscle strengthening interventions (I), knee strengthening or no intervention as comparators (C), and pain, physical function, and muscle strength as primary outcomes (O). Both controlled vocabulary (Medical Subject Headings [MeSH]) and free-text terms were used. The search strategy included terms related to patellofemoral pain, exercise therapy, hip-strengthening exercises, quadriceps strengthening, knee-strengthening exercises, pain measurement, recovery of function, and muscle strength. The complete search strategy used for PubMed is presented in Table 1, whilst the full search strategies for the remaining databases are provided in Appendix A.

### 2.2. Eligibility Criteria

Two reviewers (C.C.-M. and P.G.F.) independently screened the titles and abstracts of all records identified through the systematic search to determine their eligibility. Studies considered potentially relevant were retrieved in full text and assessed against the predefined inclusion and exclusion criteria. Disagreements were resolved through discussion, and where consensus could not be reached, a third reviewer (J.L.M.M.) was consulted.

Studies were included if they met the following criteria: (1) published in English up to 31 December 2025; (2) randomised controlled trials (RCTs); (3) participants aged 14–65 years with a clinical diagnosis of patellofemoral pain syndrome, or participants presenting with patellofemoral/anterior knee pain consistent with PFPS according to the eligibility criteria used by the original study; (4) interventions based on isolated or combined strengthening of the hip musculature, including the hip abductors, external rotators, extensors, flexors, and/or adductors, compared with knee strengthening (quadriceps and/or hamstring exercises) or no intervention; and (5) assessment of at least one of the following outcomes using validated instruments: pain, physical function, or muscle strength.

Studies were excluded if they were systematic reviews (with or without meta-analysis), study protocols or pilot studies; included participants with concomitant knee conditions; evaluated interventions not based on exercise therapy; or did not report pain, physical function or muscle strength as outcome measures (Appendix B).

### 2.3. Data Extraction and Quality Assessment

Two reviewers (C.C.-M. and P.G.F.) independently extracted the data using a standardized form adapted from the Cochrane Data Collection Form for Intervention Reviews. The following information was collected from each study: first author, year of publication, country, sample size, participant characteristics, intervention and comparator characteristics, duration of the intervention, outcome measures, and main findings. Any disagreements were resolved by consensus or, where necessary, by consultation with a third reviewer (J.L.M.M.). Duplicate records were identified and removed using Mendeley Reference Manager (version 2.100 Elsevier, London, UK).

Methodological quality was assessed independently by two reviewers using the Physiotherapy Evidence Database (PEDro) scale [29]. PEDro scores range from 0 to 10, with higher scores indicating better methodological quality. Studies were classified as excellent (9–10), good (6–8), fair (4–5) or poor (<4) according to their total PEDro score.

Risk of bias was independently evaluated using the Cochrane Risk of Bias 2 (RoB 2) tool for randomized controlled trials [30]. Overall agreement between reviewers was assessed using Cohen’s kappa coefficient (κ).

The certainty of evidence for each outcome was assessed using the Grading of Recommendations Assessment, Development and Evaluation (GRADE) approach [31]. Certainty was evaluated across the domains of risk of bias, inconsistency, indirectness, imprecision, and publication bias. A quantitative meta-analysis was not performed because the included studies showed substantial clinical and methodological heterogeneity. Differences were observed in participant characteristics and PFPS definitions, target hip and knee musculature, exercise modalities and dosage, intervention duration and supervision, comparator conditions, and outcome measurement instruments. In addition, some studies did not report complete pre- and post-intervention data, limiting the availability of sufficiently comparable data for quantitative synthesis. Therefore, the findings were synthesised narratively to preserve the clinical and methodological differences between studies rather than generate a potentially misleading pooled estimate. All GRADE assessments were conducted independently by two reviewers, with disagreements resolved through discussion or consultation with a third reviewer where necessary.

## 3. Results

### 3.1. Study Selection

**Figure 1:**
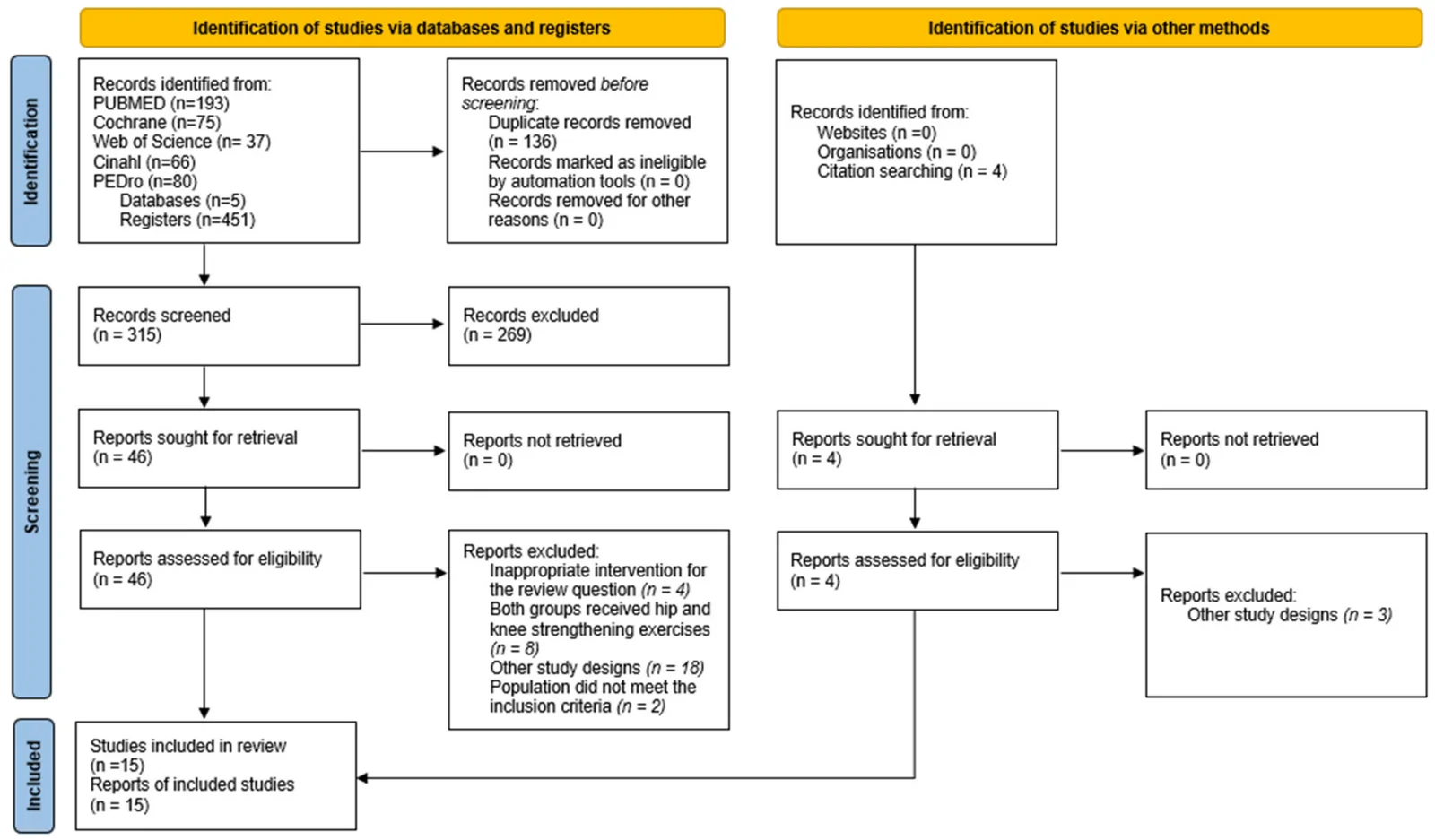
Flow diagram of the article selection process.

The literature search identified 455 potentially eligible records, including 451 retrieved through electronic database searches and 4 identified by screening the reference lists of relevant studies and systematic reviews. After removing duplicates, titles and abstracts were screened, followed by an assessment of the full text of potentially eligible articles. A total of 15 randomized controlled trials met the inclusion criteria and were included in this systematic review. The study selection process is presented in the PRISMA 2020 flow diagram (Figure 1).

### 3.2. Characteristics of the Included Studies

The main characteristics of the included studies are summarized in Appendix C, including participant characteristics, intervention protocols and outcome measures.

The 15 randomized controlled trials evaluated the effects of isolated or combined hip muscle strengthening compared with knee strengthening or no intervention in individuals with patellofemoral pain syndrome. The studies were conducted across North America, South America, Europe, Asia and the Middle East, providing broad geographical representation. Overall, 1002 participants were included, with mean ages ranging from 20 to 44 years. Women accounted for 78.2% of the study population (784 participants), whilst men represented 21.8% (218 participants).

Regarding the interventions, nine studies evaluated isolated strengthening of the hip musculature [5,17,22,24,32,33,34,35,36], whereas six studies investigated combined hip- and knee-strengthening programmes [15,16,37,38,39,40]. Hip-focused interventions primarily targeted the hip abductors, external rotators, and extensors, with some programmes also including hip flexors and adductors. Exercise modalities included isolated muscle strengthening, functional weight-bearing exercises, closed-kinetic-chain exercises, core stabilisation, and stretching. Common exercises included hip abduction and external rotation, step exercises, lunges, squats, and other functional tasks. Resistance was provided using elastic bands or free weights when reported [24,33,35,36]. Comparison programmes mainly focused on quadriceps strengthening, although some also included hamstring exercises, stretching, or core stabilisation, while several studies used no-intervention control groups [5,15,16,17,32,33,34,35,36,37,38,39,40].

Comparison groups mainly performed quadriceps strengthening exercises [5,15,16,17,32,33,34,35,36,37,38,39,40], although one study additionally included hamstring strengthening [16], whilst several studies used a no-treatment control group [16,22,24,32,34,38]. Some rehabilitation programmes also incorporated stretching [32] or core stabilization exercises [17,35,40]. The duration of the intervention ranged from 4 to 12 weeks, with 2–5 sessions per week, with three sessions per week being the most common protocol.

Pain, physical function and muscle strength were assessed using standardized outcome measures. Pain was most frequently assessed using the Visual Analogue Scale (VAS), reported in 12 of the 15 studies [5,17,22,24,32,33,34,35,37,38,39,40], although the Numeric Pain Rating Scale (NPRS), Knee Injury and Osteoarthritis Outcome Score (KOOS) and Verbal Rating Scale (VRS) were also used. Functional outcomes were primarily assessed using the Lower Extremity Functional Scale (LEFS), the Anterior Knee Pain Scale (AKPS) and the Single-Leg Hop Test (SLHT), whilst muscle strength was evaluated using isometric or isokinetic dynamometry. Hansen et al. [40] and Şahin et al. [35] reported changes without providing pre- and post-intervention values, whilst Razhegui et al. [34] evaluated muscle strength only.

### 3.3. Pain

Pain was assessed in 12 of the 15 included studies, predominantly using the Visual Analogue Scale (VAS), although the Numeric Pain Rating Scale (NPRS), Knee Injury and Osteoarthritis Outcome Score (KOOS) and Verbal Rating Scale (VRS) were also used [5,15,17,22,24,32,33,34,35,36,37,38,39,40,41].

Most studies reported significant reductions in pain following exercise-based interventions. Nine studies found greater pain reductions after programmes incorporating hip strengthening, either performed in isolation or combined with knee strengthening, than after the comparator interventions [5,15,17,22,24,32,33,34,35]. In most of these studies, pain reduction exceeded 2 points on the VAS, corresponding to the minimum clinically important difference [36].

Among studies including a no-treatment control group, both hip- and knee-strengthening interventions resulted in greater pain reductions than no intervention [16,38,39]. Ferber et al. [37] reported similar post-intervention pain levels between groups, although participants allocated to hip strengthening achieved symptom improvement approximately one week earlier. Hansen et al. [40] and Hott et al. [41] reported no significant differences between groups.

### 3.4. Physical Function

Physical function was assessed in all 15 included studies using several validated outcome measures, including the Lower Extremity Functional Scale (LEFS), the Anterior Knee Pain Scale (AKPS), the Single-Leg Hop Test (SLHT), the Knee Injury and Osteoarthritis Outcome Score (KOOS), the Western Ontario and McMaster Universities Osteoarthritis Index (WOMAC), and the Lysholm Scale [5,15,16,17,24,33,35,37,38,39,40,41].

LEFS was reported in four studies [5,15,16,17]. All reported improvements following the interventions, and exercise-based programmes were superior to no treatment when this comparator was included. Fukuda et al. [15] reported greater improvements following combined hip and knee strengthening than knee strengthening alone.

AKPS was evaluated in seven studies [15,16,33,35,37,39,40,41]. Three studies reported significant improvements over time with no differences between groups [37,40,41], whilst three reported greater improvements following hip strengthening than knee strengthening [15,35] or closed kinetic chain exercises [33]. In studies including a no-treatment control group, both exercise interventions produced better outcomes than no treatment [37,39].

Similar findings were observed with the other functional measures. Two studies reported better SLHT performance following hip strengthening [15,17], whereas Fukuda et al. [16] found no differences between the groups. One study reported better WOMAC scores following hip strengthening [24], whilst KOOS scores were comparable between groups [40]. The Lysholm Scale was assessed in one study, with both exercise groups showing greater improvement than the no-treatment control group [38].

### 3.5. Muscle Strength

Muscle strength was assessed in 10 of the 15 included studies, primarily using isometric or isokinetic dynamometry to evaluate hip and knee muscle strength [5,17,22,24,32,33,34,35,37,39,40,41].

Eight studies reported greater improvements in hip muscle strength following interventions that incorporated hip strengthening [5,17,22,24,34,35,37,39]. Four studies also reported comparable gains in selected hip muscle groups, including the abductors, adductors, flexors and external rotators, following knee-strengthening programmes [5,17,37,39].

Three studies found no significant differences between groups in muscle strength following hip- or knee-focused exercise programmes [32,33,40]. In contrast, studies including a no-treatment control group reported greater strength gains following exercise-based interventions than after no intervention. Razhegui et al. [34] observed significant improvements across all assessed hip muscle groups following hip strengthening, whereas no significant changes in knee extensor strength were found in either group.

### 3.6. Methodological Quality and Risk of Bias

Methodological quality was assessed using the PEDro scale (Table 2). PEDro scores ranged from 4 to 8, with a mean score of 7, indicating overall moderate-to-high methodological quality across the included studies. As expected for exercise-based interventions, the lowest scores were observed for participant and therapist blinding (items 5 and 6). Inter-rater agreement for the PEDro assessment was excellent (Cohen’s κ = 0.865).

**Figure 2:**
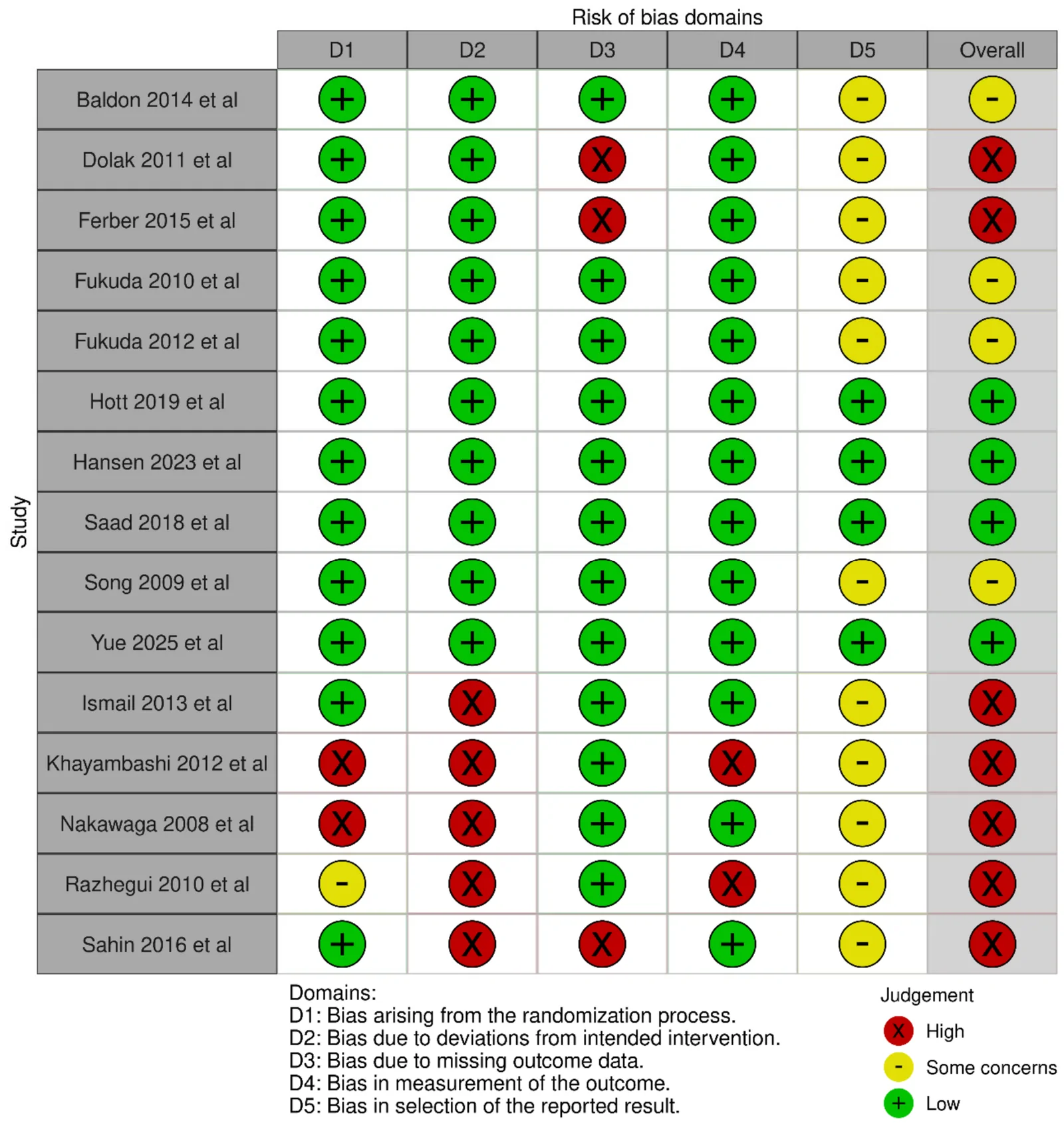
Assessment of risk of bias: summary of individual studies [5,15,16,17,20,22,24,32,33,34,35,37,38,39,40,41].

**Figure 3:**
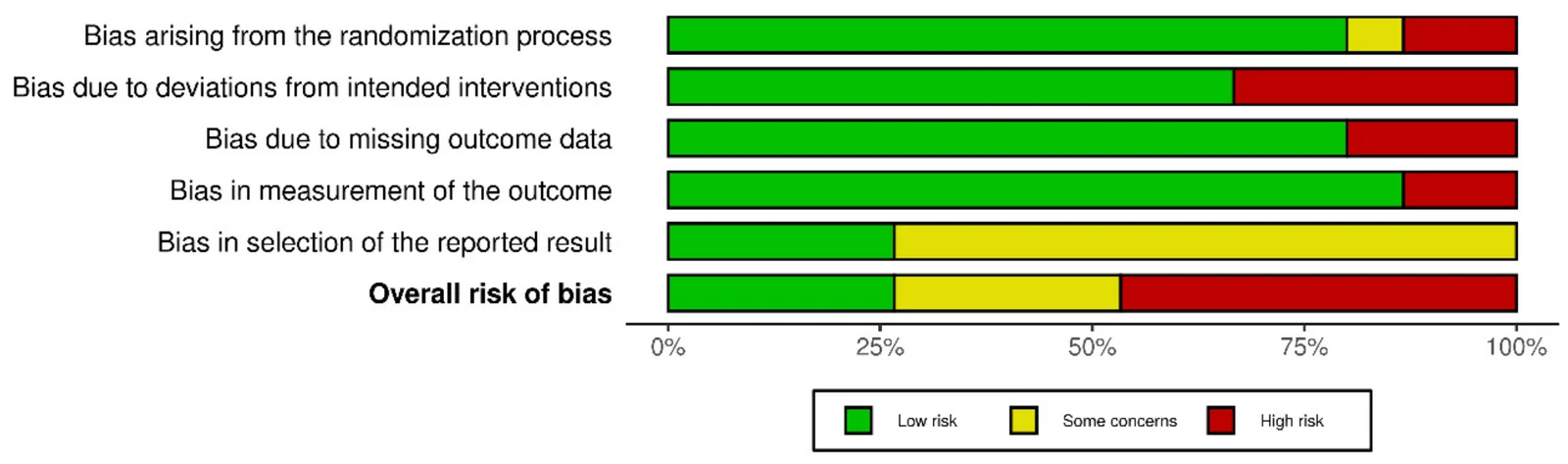
Assessment of risk of bias: pooled results of the assessment.

Risk of bias was assessed using the Cochrane Risk of Bias 2 (RoB 2) tool (Figure 2 and Figure 3). Overall, 7 studies (46%) were judged to have a high risk of bias, 4 (27%) raised some concerns, and 4 (27%) were considered to have a low risk of bias. Agreement between reviewers was good (Cohen’s κ = 0.755).

Studies using intention-to-treat analyses generally demonstrated a lower risk of bias in the randomization process and missing outcome data, although some concerns remained regarding outcome measurement and selective reporting. In contrast, studies analyzed according to per-protocol analyses showed a less favorable risk-of-bias profile, particularly in the domains related to deviations from the intended intervention, selective reporting, and the overall risk-of-bias judgement.

### 3.7. Certainty of Evidence

The certainty of evidence was assessed using the GRADE approach (Table 3). Overall, the certainty of evidence was rated as moderate for all three primary outcomes.

For pain, the certainty of evidence was downgraded due to methodological limitations identified in several studies and some variability in treatment effects across trials, despite most studies reporting clinically relevant improvements following exercise interventions.

For physical function, the certainty of evidence was also considered moderate. Although functional improvements were consistently observed, differences between groups were generally small, and heterogeneity in the outcome measures contributed to inconsistency across studies.

Similarly, the certainty of evidence for muscle strength was rated as moderate. Hip-strengthening interventions consistently improved proximal muscle strength; however, differences in the methods used to assess muscle strength introduced indirectness, resulting in a downgrading of the certainty of evidence.

## 4. Discussion

This systematic review synthesised the evidence from randomised clinical trials that assessed the effects of hip muscle strengthening, either alone or in combination with knee strengthening, compared with knee strengthening alone or no intervention in people with PFPS. Overall, the results indicate that exercise-based interventions can improve pain, physical function and muscle strength, reinforcing current recommendations that position exercise-based interventions as a cornerstone of PFPS treatment [1,2].

Although most exercise programmes produced clinically relevant improvements, those incorporating hip muscle strengthening generally showed greater reductions in pain and larger improvements in proximal muscle strength than knee-focused programmes, whereas functional outcomes were generally comparable. These findings do not support replacing knee strengthening with hip strengthening but rather integrating both into individualised rehabilitation programmes that simultaneously address proximal neuromuscular deficits and local factors related to the knee.

Our results are consistent with those reported by Rogan et al. [13], Alammari et al. [1], and Halabi et al. [14], who also concluded that hip muscle strengthening provides additional benefits in the management of PFPS. Unlike previous reviews, the present systematic review offers the first integrated synthesis of the effects of hip muscle strengthening on pain, physical function, and muscle strength, while also incorporating a comprehensive assessment of methodological quality (PEDro), risk of bias (ROB 2), and certainty of evidence (GRADE). Furthermore, it includes the most recent randomized controlled trials published up to December 2025, providing a more comprehensive and clinically relevant synthesis to inform exercise prescription.

Pain was the variable that showed the most consistent results. Most studies demonstrated significant improvements following exercise programmes. These findings are consistent with those of Rogan et al. [13], Alammari et al. [1] and Halabi et al. [14], who observed greater reductions in pain when programmes incorporated hip muscle strengthening. The present review reinforces these conclusions by including more recent clinical trials and confirming that exercise remains superior to no intervention.

When comparing the different strategies, hip muscle strengthening showed a tendency to produce greater reductions in pain. Of the eight studies that evaluated this intervention [5,17,22,24,37,39,40,41], six achieved clinically relevant improvements of more than 2 points on the VAS [17]. Although knee-focused programmes, which were evaluated in 12 studies [5,15,16,17,32,34,35,37,38,39,40,41], also reduced pain, only three demonstrated significant differences at the end of treatment [17,38,39], which could indicate a clinical advantage of the proximal approach. It is worth noting that the control group in the study by Ismail et al. [33], which involved functional closed-kinetic-chain exercises primarily targeting the quadriceps, also showed significant improvements compared with baseline.

Combined hip and knee strengthening programmes, evaluated in six studies [15,16,32,34,35,38], showed consistently positive results, supporting an approach that combines the treatment of proximal and local factors. Furthermore, some studies reported a more rapid reduction in pain following hip strengthening [5,37], which could promote adherence and facilitate functional recovery during the early stages of rehabilitation.

Except for Hansen et al. [40], all studies included at least one supervised session per week. Supervision can improve adherence, ensure the exercises are performed correctly and facilitate an appropriate progression of therapeutic loads [1,42]. The absence of differences between groups in Hansen et al. [40] may be related to the lack of supervision or to the inclusion of functional exercises that may have activated the hip stabilising muscles, particularly the gluteus medius.

Regarding programme duration, most studies [5,17,22,24,32,33,35,37,38,39,41] implemented programmes lasting 6 to 8 weeks with an average frequency of three sessions per week, which is sufficient to achieve clinically relevant changes. However, some studies observed improvements after just four weeks [15,16,34], whilst a 12-week intervention showed no differences between groups [40], suggesting that, within the 4- to 12-week range observed across studies, clinically relevant improvements were reported with different intervention durations, although the optimal duration remains uncertain.

Compared with pain, functional outcomes showed greater heterogeneity, probably due to the diversity of assessment tools used. Nevertheless, the evidence indicates that hip-strengthening, knee-strengthening or combined programmes improve physical function, without any consistent superiority of one strategy over another.

These findings are consistent with those described by Rogan et al. [13], Alammari et al. [1] and Halabi et al. [14], who also concluded that functional recovery depends primarily on undertaking a progressive exercise programme, rather than on the specific muscle group being strengthened.

The LEFS, assessed in four studies [5,15,16,17], showed improvements exceeding the minimum clinically important difference in the hip-strengthening groups and in the combined programmes. Furthermore, three studies [5,16,17] also observed significant improvements in the knee-strengthening groups, which were greater than those in the no-intervention group when this was included [16]. Similarly, most studies using the AKPS [15,16,33,35,37,39,40,41] reported functional improvements following the intervention. Four of these [15,16,33,35] found greater benefits with combined programmes, whilst the remaining studies did not observe consistent differences between the interventions [37,39,40,41].

Similar results were obtained with other functional scales. Baldon et al. [17] and Fukuda et al. [15] reported better performance on the SLHT following hip-strengthening or combined programmes, whilst scores obtained using the KOOS and Lysholm scales were comparable across interventions [24,38,40]. Taken together, these results suggest that functional recovery depends primarily on therapeutic exercise, although combining hip and knee strengthening may provide additional benefits by simultaneously targeting the proximal and distal musculature.

Unlike function, the results relating to muscle strength were more consistent and showed specific adaptations depending on the type of intervention. Overall, programmes incorporating hip muscle strengthening produced greater increases in the strength of the hip abductors, external rotators and extensors. These muscle groups have been associated with proximal neuromuscular control and lower-limb movement patterns in individuals with PFPS [13,14]. However, as the included studies primarily assessed clinical outcomes and muscle strength rather than patellofemoral joint loading or lower-limb kinetics and kinematics, these findings should not be interpreted as direct evidence of reduced patellofemoral loading or improved movement mechanics.

These findings expand upon the evidence provided by the reviews by Rogan et al. [13], Alammari et al. [1] and Halabi et al. [14], which focused primarily on pain and function, by offering a specific synthesis of the changes in muscle strength resulting from different strengthening strategies.

Seven studies reported greater gains in proximal muscle strength in programmes incorporating hip strengthening than in programmes focusing on the quadriceps [5,17,32,34,35,37,39]. Of these, four evaluated programmes involving isolated hip strengthening [5,17,37,39] and three evaluated combined programmes [32,34,35]. Furthermore, Khayambashi et al. [22] and Yue et al. [24] demonstrated significant improvements in all muscle groups compared with the non-intervention group, reinforcing the clinical efficacy of strength training.

In the hip external rotators, three studies found greater increases following hip-strengthening or combined programmes [34,35,39], whilst seven others observed no differences between interventions [5,17,32,33,37,40,41], although all showed improvements compared with baseline. Taken together, these findings suggest that appropriately dosed exercise programmes can produce clinically relevant improvements in strength and function, although the specific biomechanical mechanisms underlying these effects cannot be established from the present evidence [13,14].

Finally, some studies observed increases in hip flexor strength following proximal strengthening programmes [17,34], whilst the rest found no differences, probably due to the indirect activation of the rectus femoris during knee extension exercises. Similarly, the absence of differences between groups in knee extensor strength, even in hip-focused interventions, could be explained by the use of closed-kinetic-chain exercises [33], which promote global activation of the lower limb, as well as by neuromuscular adaptations that allow for increased strength even in muscles not specifically trained [43].

The interpretation of the results must also take into account methodological quality, risk of bias and the certainty of the evidence. Assessment using the PEDro scale revealed moderate-to-high overall methodological quality, with strengths in randomisation, comparability between groups and reporting of results. However, limitations inherent to exercise interventions were common, such as the absence of blinding, small sample sizes, variability in protocols, and some shortcomings in concealed allocation or the handling of missing data, factors that may compromise internal validity [29,44].

The ROB 2 assessment confirmed these limitations. Studies that used intention-to-treat analyses presented a lower risk of bias than those based on per-protocol analyses, although some concerns remained regarding the randomisation process, deviations from the intended intervention, outcome measurement and the selection of reported outcomes [30,45,46].

Overall, the evidence supports the effectiveness of programmes that include hip muscle strengthening to improve pain, function and muscle strength in people with PFPS. However, the certainty of the evidence was moderate due to the risk of bias, heterogeneity in protocols and assessment tools, and the imprecision of some outcomes; therefore, future research could alter the current estimates [31,47].

## 5. Clinical Implications

The results of this review support the use of therapeutic exercise programmes in the conservative management of people with PFPS. In clinical practice, the exercise prescription should focus not only on strengthening the knee muscles but also on specific training of the proximal hip muscles, integrating both strategies into progressive and individualised programmes. Furthermore, treatment supervision, appropriate progression of exercise loads and patient adherence are fundamental to optimising clinical outcomes.

## 6. Limitations and Strengths

The results of this review must be interpreted in light of several limitations. The substantial heterogeneity across the included studies limits direct comparison and the interpretation of the overall magnitude of treatment effects. This heterogeneity involved participant characteristics and PFPS definitions, intervention content and dosage, comparator conditions, supervision, follow-up duration, and outcome measurement instruments. Consequently, although a consistent direction of benefit was observed for exercise-based interventions, the magnitude of the effects and the relative contribution of specific strengthening strategies cannot be established with precision. The absence of a quantitative synthesis should therefore be considered when interpreting the overall strength and generalisability of the evidence.

Furthermore, adherence to treatment was not assessed in a standardised manner and some studies did not analyse all the variables of interest, which limits the consistency of the available evidence. Finally, the exclusive inclusion of studies published in English may have introduced a language bias.

An additional source of clinical heterogeneity was the variability in the criteria used to define PFPS across the included trials. Although the included studies recruited participants with patellofemoral or anterior knee pain consistent with the condition under investigation, some trials used explicit clinical diagnostic criteria whereas others relied primarily on symptom-based definitions. This variability may have influenced the clinical characteristics of the included populations and should be considered when interpreting the generalisability of the findings.

In addition, most included studies focused on clinical outcomes and muscle strength, with limited direct assessment of patellofemoral joint loading, lower-limb kinematics, or kinetics. Therefore, the biomechanical mechanisms potentially underlying the observed clinical improvements cannot be established from the present evidence and should be considered as plausible explanations rather than demonstrated effects.

Among the strengths of this review, we included only randomised controlled trials and incorporated a systematic assessment of methodological quality, risk of bias, and certainty of evidence, providing a critical and robust synthesis of the available evidence. Furthermore, the structured narrative synthesis of the effects on pain, function and muscle strength offers a clinically relevant perspective to guide the prescription of exercise in patients with PFPS.

## 7. Future Research Directions

Future research should focus on developing clinical trials with more standardised intervention protocols and assessment tools, particularly for variables relating to function and muscle strength, in order to improve comparability between studies. It is also necessary to assess the medium- and long-term effects of strength-training programmes and to identify the influence of individual factors, such as gender, age, level of physical activity or biomechanical characteristics, which will enable progress towards more personalised treatment strategies.

Future research should also explore biomechanics-informed, patient-specific rehabilitation strategies. Recent computational approaches have combined gait-derived biomechanical information with finite element analysis, machine learning, and parametric design to optimise personalised assistive devices according to individual loading patterns [48,49]. For example, computational frameworks have been developed to adapt lattice-based devices to gait-induced plantar pressure distributions and to optimise parametrised cushioning insoles using finite element and machine-learning approaches [48,49]. Although these studies were not specifically focused on PFPS treatment, they illustrate a potential precision-rehabilitation framework in which patient-specific biomechanical characteristics could inform the selection and optimisation of rehabilitation strategies. Similar approaches could be investigated in PFPS to determine whether biomechanical profiling can help identify clinically meaningful subgroups and guide more individualised exercise and adjunctive interventions.

## 8. Conclusions

The findings of this systematic review indicate that exercise-based interventions can improve pain, physical function, and muscle strength in individuals with PFPS. Programmes incorporating hip muscle strengthening may provide additional benefits, particularly for pain reduction and proximal muscle strength, although functional outcomes appear broadly comparable across strengthening strategies. However, the heterogeneity of participant characteristics, intervention protocols, outcome measures, and diagnostic criteria, together with the risk of bias identified in several included studies, limits the certainty with which the relative effectiveness of specific strengthening strategies can be established. Therefore, the current evidence is insufficient to determine whether one strengthening strategy provides a clear overall advantage over others. Future high-quality randomised controlled trials using standardised diagnostic criteria, intervention protocols, and outcome measures are warranted to determine the optimal and most individualised exercise prescription for PFPS.

## Figures and Tables

**Table 1:** Search strategy—PubMed database.

Search Strategy	Terms and Search Operators	Results Obtained
PubMed	15 January 2026	
#1	(“patellofemoral pain” OR “anterior knee pain” OR “chondromalacia patella”)	193
#2	(“Exercise Therapy” [Mesh] OR “Exercise Therapy/methods” [Mesh] OR “Physical Therapy Modalities” [Mesh] OR “Patellofemoral Pain Syndrome/rehabilitation” [Mesh] OR “Hip Physiology” [Mesh] OR “Combined Modality Therapy” [Mesh] OR “hip exercises” OR “hip-strengthening exercises”)	
#3	(“Quadriceps Muscle physiology/physiopathology” [Mesh] OR “Quadriceps Muscle” [Mesh] OR “quadriceps strengthening exercise” OR “quadriceps strengthening” OR “Knee strengthening exercise” OR “Knee stretching exercises” OR “Knee Stabilising Exercises” OR “no treatment” OR “control group”)	
#4	(“Treatment outcome” [Mesh] OR “Pain Measurement” [Mesh] OR “Recovery of Function” [Mesh] OR “function” OR “functionality” OR “muscle strength” OR “strength”)	
#5	#1 AND #2 AND #3 AND #4	

**Table 2:** Methodological quality measured using the PEDro scale.

Study	1	2	3	4	5	6	7	8	9	10	11	Total
Baldon et al. 2014 [17]	+	+	+	+	−	−	+	+	+	+	+	8/10
Dolak et al. 2011 [5]	+	+	+	+	−	−	+	−	+	+	+	7/10
Ferber et al. 2015 [37]	+	+	+	+	−	−	+	−	+	+	+	7/10
Fukuda et al. 2010 [16]	+	+	+	+	−	−	+	+	+	+	+	8/10
Fukuda et al. 2012 [15]	+	+	+	+	−	−	+	+	+	+	+	8/10
Hott et al. 2019 [41]	+	+	+	+	−	−	+	+	+	+	+	8/10
Ismail et al. 2013 [33]	+	+	+	+	−	−	+	+	−	+	+	7/10
Khayambashi et al. 2012 [22]	+	−	−	+	−	−	−	+	−	+	+	4/10
Hansen et al. 2023 [40]	+	+	+	+	−	−	+	+	+	+	+	8/10
Nakagawa et al. 2008 [32]	+	+	+	+	−	−	+	+	−	+	+	7/10
Razhegui et al. 2010 [34]	+	+	−	−	−	−	+	+	−	+	+	5/10
Saad et al. 2018 [39]	+	+	+	+	−	−	+	+	+	+	+	8/10
Song et al. 2009 [38]	+	+	+	+	−	−	+	+	+	+	+	8/10
Yue et al. 2025 [24]	+	+	+	+	−	−	+	−	−	+	+	6/10
Sahin et al. 2016 [35]	+	+	+	+	−	−	+	+	−	+	+	7/10

“+” = criterion met, “−“ = criterion not met. 1. Eligibility criteria and source (this item is not included in the total score), 2. Random allocation, 3. Allocation concealment, 4. Baseline comparability, 5. Participant blinding, 6. Therapist blinding, 7. Assessor blinding, 8. Adequate follow-up (>85%), 9. Intention-to-treat analysis, 10. Statistical comparisons between groups, 11. Presentation of point estimates and measures of variability.

**Table 3:** GRADE assessment.

Certainty Assessment							Patients		Certainty	Importance
Studies	Study Design	Risk of Bias	Inconsistency	Indirectness	Imprecision	Other	Hip Exercise	Control		
Outcome: Pain					Hip strengthening versus control					
15	Randomised trials	serious	Serious	Not serious	Not serious	No	459 (46%)	543 (54%)	⨁⨁⨁◯	Critical
									Moderate	
Outcome: Physical Function					Hip strengthening versus control					
12	Randomised trials	Serious	Serious	Not serious	Not serious	No	427 (46%)	511 (54%)	⨁⨁⨁◯	Critical
									Moderate	
Outcome: Muscle Strength					Hip strengthening vs. Control					
10	Randomised trials	Serious	Not serious	Serious	Not serious	No	370 (48%)	401 (52%)	⨁⨁⨁◯	important
									Moderate	

Note: Control group = knee strengthening or no intervention. ⨁ = certainty of evidence; ◯ = certainty downgraded by one level.

## Data Availability

The data and analyses generated during this study are available from the corresponding author upon reasonable request.

## Supplementary Materials

The following supporting information can be downloaded at: https://www.mdpi.com/article/10.3390/muscles5030066/s1, Table S1: PRISMA 2020 Checklist.

## Appendix A

**Table A1:** Terms Used in the Search Equations by Database.

Search Strategy	Terms and Equations	Results
CINAHL	22 January 2026	66
S1	XB (MH “Patellofemoral Pain Syndrome”) OR XB (“anterior knee pain”) OR XB (“patellofemoral pain”) OR XB (“chondromalacia patella”)	
S2	“hip strengthening” OR MH “Combined Modality Therapy” OR “hip exercis*” OR “hip muscles” OR “hip physiology”	
S3	MH “Quadriceps Muscles” OR MH “Control Group” OR “control group” OR “quadriceps strength*” OR “knee strengthen*” OR “quadriceps exercis*”	
S4	(MH “Pain Measurement” OR “pain”) OR (“muscle strength” or “strength”) OR “function”	
S5	S1 AND S2 AND S3 AND S4	
Cochrane	1 February 2026	75
#1	MeSH descriptor: [Patellofemoral Pain Syndrome] expand all trees OR (“patellofemoral pain” OR “anterior knee pain” OR “chondromalacia patellae”):ti,ab,kw	
#2	(“hip exercises”) OR (“hip muscles”) OR (“hip strengthening”) OR MeSH descriptor: [Combined Modality Therapy] expand all trees	
#3	MeSH descriptor: [Quadriceps Muscle] explode all trees OR (“quadriceps strengthening”) OR (“quadriceps exercises”) OR (“knee strengthening”) OR MeSH descriptor: [Control Groups] explode all trees OR (“control group”)	
#4	MeSH descriptor: [Muscle Strength] expand all trees OR (“pain”) OR MeSH descriptor: [Recovery of Function] expand all trees	
#5	#1 AND #2 AND #3 AND #4	
Web of Science	7 February 2026	37
#1	TI=(“patellofemoral pain” OR “patellofemoral pain syndrome” OR “anterior knee pain” OR “chondromalacia patella*”)	
#2	TS=(“hip exercise*” OR “hip strengthening” OR “hip muscles”)	
#3	TS=(“quadriceps strengthening” OR “quadriceps exercise*” OR “knee strengthening” OR “control group”)	
#4	TS=(“pain” OR “function” OR “strength”)	
#5	#1 AND #2 AND #3 AND #4	
PEDro	10 February 2026	80
Abstract & Title	“patellofemoral pain” “hip”	
Therapy	strength training	
Body Part	lower leg or knee	
Subdiscipline	musculoskeletal	
When searching:	Match all search terms (AND)	

# = search strategy set number. * = truncation symbol used to retrieve terms with different word endings.

## Appendix B

**Table A2:** PICO Framework Used to Define Inclusion and Exclusion Criteria.

PICO Categories	Inclusion	Exclusion
P (Population)	Patients aged between 14 and 65 years diagnosed with or exhibiting symptoms of PFPS, provided they experienced anterior knee pain when engaging in physical activities or whilst sitting.	Patients with PFPS, alongside other underlying knee conditions.
I (Intervention)	Exercise protocol for the hip muscles, both in isolation and in combination with knee exercises.	Non-exercise-based intervention.
C (Comparator)	Knee-strengthening programme or no intervention.	Control group with hip exercises or another type of training.
O (Outcome)	Studies that measured pain, function and/or strength using standardised scales or measurement tools.	Studies that measured other variables.
s (Study design)	RCTs published in English up to 31 December 2025.	Systematic reviews with and without meta-analyses, protocol registries and pilot studies.

## Appendix C

**Table A3:** Characteristics and Results of the Included Studies.

Author/Year	Country	Intervention Analysis	Sample Size	Age (SD)	Gender M/F	Intervention Characteristics	Intervention Duration	Measurement Tool	Results						
Baldon et al. 2014 [17]	Brazil	ITT	IG: 15 CG: 16	IG: 27.7 ± 3.2 CG: 21.3 ± 2.6	M 0/31	IG: hip + core and functional stabilisation exercises CG: knee + stretching	8 weeks 3 days a week (under supervision)	Pain: EVA Function: LEFS, SLTH. Strength: isokinetic dynamometry.	PAIN VAS: Pre-IG: 6.6 ± 1.1 Post: 1.4 ± 1.4 CG Pre: 6.1 ± 1.8 Post: 3.1 ± 3.2		FUNCTION LEFS: IG Pre: 55.4 ± 12.8 Post: 74.3 ± 4.6 CG Pre: 57.6 ± 7.2 Post: 70.6 ± 8.0	STRENGTH			
												Triple hop test/cm	Dynamometry (Nm/kg):		
												IG Pre: 336.4 ± 34.8 Post: 375.3 ± 48.3 CG Pre: 325.1 ± 82.4 Post: 330.1 ± 72.5	FLEX (+)/EXT (−) HIP: IG Pre: 52.5 ± 14.6 Post: 67.5 ± 14.0 CG Pre: 46.9 ± 9.3 Post: 45.4 ± 12.3	ADD (+)/ABD (−) HIP: IG Pre: 23.5 ± 6.2 Post: 12.3 ± 6.9 CG Pre: 17.1 ± 4.3 Post: 15.4 ± 4.6	RE (ECCENTRIC): IG Pre: 0.8 ± 0.1 Post: 0.7 ± 0.1 CG Pre: 0.8 ± 0.1 Post: 0.8 ± 0.1
Dolak et al. 2011 [5]	USA	ITT	IG: 17 CG: 16	I: 25 ± 5 CG: 26 ± 6	M 0/33	IG: hip + functional weight-bearing exercises + stretching CG: knee + functional weight-bearing exercises + stretching	8 weeks 3 days a week (1 supervised)	Pain: EVA Function: LEFS Strength: isometric dynamometry (Nm/kg)	PAIN EVA: Pre-IG: 4.6 ± 2.5 Post: 2.4 ± 2.8 CG Pre: 4.2 ± 2.3 Post: 2.6 ± 2.0		FUNCTION LEFS: IG Pre: 59 ± 12 Post: 70 ± 10 CG Pre: 54 ± 12 Post: 65 ± 13	STRENGTH Dynamometry (Nm/kg)			
												HIP ABDUCTORS: IG Pre: 5.2 ± 1.5 Post: 6.6 ± 0.9 CG Pre: 5.7 ± 2.2 Post: 6.2 ± 1.8	RE: IG Pre: 2.1 ± 0.7 Post: 2.7 ± 0.7 CG Pre: 2.1 ± 1.0 Post: 2.2 ± 0.7		QUADRICEPS: IG Pre: 6.1 ± 2.6 Post: 7.0 ± 1.4 CG Pre: 6.3 ± 2.1 Post: 6.6 ± 1.9
Ferber et al. 2015 [37]	Canada/USA	ITT	IG: 111 CG: 88	IG: 30 ± 4.3 CG: 28.8 ± 3.51	H and M 66/133	IG: hip + core CG: knee	6 weeks 3 days a week (under supervision) Home exercises (6 days a week)	Pain: VAS Function: AKPS Strength: isometric dynamometry (Nm/kg)	PAIN VAS: Pre-intervention IG: 5.12 ± 1.66 Post: 1.96 ± 1.92 CG Pre: 4.96 ± 1.66 Post: 1.99 ± 2.05		FUNCTION AKPS: Pre-IG: 75.0 ± 9.7 Post: 87.95 ± 11.26 CG Pre: 75.62 ± 9.81 Post: 87.67 ± 10.53	STRENGTH Dynamometry (Nm/kg):			
												ABD HIP: Pre-IG: 3.21 ± 1.14 Post: 3.58 ± 1.08 CG Pre: 3.15 ± 1.19 Post: 3.41 ± 1.28	EXT HIP: IG Pre: 2.39 ± 1.01 Post: 2.66 ± 1.15 CG Pre: 2.44 ± 1.09 Post: 2.61 ± 1.18		RE: IG Pre: 1.19 ± 0.42 Post: 1.18 ± 0.45 CG Pre: 1.29 ± 0.41 Post: 1.25 ± 0.44
Fukuda et al. 2010 [16]	Brazil	ITT	IG: 23 CG: 22 CG: 25	IG: 25 ± 7 CG: 25 ± 6 CG (NT): 24 ± 7	M 0/70	IG: hip + knee + stretching CG: knee + stretching CG (NT): no intervention.	4 weeks 3 days a week (under supervision)	Pain: NPRS Function: LEFS, AKPS, SLTH	PAIN NPRS:			FUNCTION			
									Climbing stairs: Pre-IG: 5.2 ± 1.6 Post: 3.0 ± 1.8 CG Pre: 4.9 ± 2.9 Post: 3.4 ± 2.3 CG (NT) Pre: 4.9 ± 2.5 Post: 5.0 ± 2.5		Going down stairs: IG Pre: 4.9 ± 1.6 Post: 2.3 ± 1.5 CG Pre: 4.5 ± 2.8 Post: 3.5 ± 2.5 CG (NT) Pre: 4.4 ± 2.4 Post: 4.1 ± 2.3	LEFS: IG Pre: 49.1 ± 11.9 Post: 65.7 ± 13.5 CG Pre: 55.6 ± 15.9 Post: 65.6 ± 14.5 CG (NT) Pre: 48.8 ± 17.0 Post: 51.2 ± 15.1	AKPS: IG Pre: 63.9 ± 11.7 Post: 78.9 ± 16.0 CG Pre: 70.4 ± 12.5 Post: 80.6 ± 13.9 CG (NT) Pre: 63.8 ± 15.5 Post: 64.5 ± 11.1		Hop test (cm): IG Pre: 76.1 ± 33.8 Post: 91.8 ± 34.4 CG Pre: 76.1 ± 37.7 Post: 86.5 ± 32.0 CG (NT) Pre: 81.0 ± 25.5 Post: 80.3 ± 16.0
Fukuda et al. 2012 [15]	Brazil	ITT	IG: 28 CG: 26	IG: 22 ± 3 CG: 23 ± 3	M 0/54	IG: hip + knee + stretching CG: knee + stretching	4 weeks 3 days a week (under supervision)	Pain: NPRS Function: LEFS, AKPS, SLTH	PAIN NPRS:			FUNCTION			
									Climbing stairs: Pre-IG: 6.2 ± 1.1 Post 3M: 1.2 ± 1.1 CG Pre: 6.6 ± 1.2 Post 3M: 5.3 ± 1.3		Going down stairs: IG Pre: 5.8 ± 1.2 Post 3M: 1.6 ± 1.1 CG Pre: 6.4 ± 1.4 Post 3M: 5.0 ± 1.2	LEFS: IG Pre: 51.7 ± 10.4 Post 3M: 74.1 ± 5.6 Pre-CG: 49.0 ± 13.0 Post 3M: 49.4 ± 11.2	AKPS: Pre-IG: 65.9 ± 8.5 Post 3M: 85.7 ± 9.0 CG Pre: 61.8 ± 9.0 Post 3M: 64.6 ± 10.2		Hop test (cm): IG Pre: 69.9 ± 10.4 Post 3M: 85.7 ± 10.2 CG Pre: 61.7 ± 22.6 Post 3M: 69.9 ± 21.8
Hott et al. 2019 [41]	Norway	ITT	IG: 39 CG: 37 CG: 36	IG: 27.8 ± 8.6 CG: 28.5 ± 6.2 CG (NT): 26.3 ± 7.0	H and M 39/73	IG: hip CG: knee CG (NT): free physical activity	6 weeks 3 days a week (1 supervised)	Pain: VAS Function: AKPS Strength: step-down test, isometric strength	PAIN		FUNCTION AKPS: IG (Pre): 64.5 Post: 73.3 CG Pre: 67.2 Post: 74.5 CG (NT) Pre: 65.3 Post: 73.6	STRENGTH Dynamometry (isometric, N):			
									VAS (usual): IG Pre: 4.4 Post: 3.1 CG Pre: 4.3 Post: 2.6 CG (NT) Pre: 3.7 Post: 2.8	VAS (maximum): IG Pre: 6.5 Post: 5.3 CG Pre: 6.0 Post: 4.1 CG (NT) Pre: 5.8 Post: 4.7		HIP: IG Pre: 138 Post: 150 CG Pre: 126 Post: 143 CG (NT) Pre: 138 Post: 141	HIP RE: IG Pre: 111 Post: 124 CG Pre: 103 Post: 117 CG (NT) Pre: 114 Post: 119		OUTSIDE KNEE: IG Pre: 321 Post: 345 CG Pre: 317 Post: 326 CG (NT) Pre: 337 Post: 322
Ismail et al. 2013 [33]	Egypt	PP	IG: 16 CG: 16	IG: 20.8 ± 2.7 CG: 21.2 ± 3.2	M and F 9/23	IG: hip + closed kinetic chain + stretching CG: closed kinetic chain + stretching	6 weeks 3 days a week	Pain: VAS Function: Kujala score/AKPS Strength: isokinetic dynamometry (Biodex)	PAIN VAS: Pre-IG: 5.3 ± 1.6 Post: 2.0 ± 1.1 CG Pre: 4.5 ± 1.8 Post: 2.3 ± 1.1		FUNCTION Kujala: Pre-IG: 71.5 ± 7.8 Post: 85.1 ± 6.2 CG Pre: 76.4 ± 10.4 Post: 85.0 ± 6.7	STRENGTH Isokinetic dynamometry (Torque/BMI):			
												HIP ABD: Pre-IG: 1.7 ± 0.6 Post: 2.4 ± 0.8 CG Pre: 2.1 ± 0.6 Post: 2.5 ± 0.7			RE HIP: IG Pre: 0.9 ± 0.6 Post: 1.3 ± 0.6 CG Pre: 1.0 ± 0.4 Post: 1.2 ± 0.4
Khayambashi et al. 2012 [22]	Iran	PP	IG: 14 CG: 14	IG: 28.9 ± 5.8 CG: 30.5 ± 4.8	M 0/28	IG: hip CG (NT): No exercise.	8 weeks 3 days a week (Supervised)	Pain: EVA Function: WOMAC Strength: hand dynamometry	PAIN VAS: Pre-IG: 7.9 ± 1.7 Post: 1.4 ± 1.9 CG Pre: 6.6 ± 2.0 Post: 6.7 ± 2.4	FUNCTION WOMAC: IG Pre: 54.0 ± 18.1 Post: 10.7 ± 16.1 CG Pre: 55.9 ± 13.5 Post: 59.9 ± 12.6	STRENGTH Manual dynamometry (N):				
											RIGHT HIP ABD: IG Pre: 11.6 ± 2.3 Post: 15.3 ± 2.5 CG Pre: 12.3 ± 2.9 Post: 11.2 ± 2.5	Left hip ABD: IG Pre: 11.2 ± 2.7 Post: 15.9 ± 3.1 CG Pre: 12.5 ± 3.7 Post: 11.4 ± 3.1	RIGHT RE: IG Pre: 8.6 ± 2.3 Post: 11.8 ± 2.2 CG Pre: 8.9 ± 2.1 Post: 8.3 ± 2.3		LEFT RE: IG Pre: 7.0 ± 1.8 Post: 10.9 ± 2.6 CG Pre: 7.5 ± 1.6 Post: 7.3 ± 1.9
Hansen et al. 2023 [40]	Denmark	ITT	IG: 100 CG: 100	IG: 27.2 ± 6.7 CG: 27.2 ± 6.3	H and M 62/138	IG: hip CG: knee	12 weeks 3 days a week (Unsupervised)	Pain/Function: AKPS and KOOS. Additional pain: VRS Strength: isometric dynamometry	This study reports changes, not direct pre-post values. PAIN VRS: Post-intervention control group: 1.8 (1.8) CG Post: 1.9 (2.2)	KOOS: - Pain: IG: +6.4 CG: +9.3 - Function: IG: +4.8 CG: +5.8 - Quality of life: IG: +11.9 CG: +10.7	AKPS: IG Post: +7.0 (5.2–8.9) CG Post: +7.6 (5.8–9.5)	STRENGTH Isokinetic dynamometry (N):			
												ABD HIP: IG: +13.3 CG: +13.8 ADD: IG: +16.2 CG: +10.9	RI: IG: +10.6 CG: +9.3 RE: IG: +4.9 CG: +4.7		QUADRICEPS: IG: +32.9 CG: +33.7 HAMSTRINGS: IG: +41.8 CG: +37.8
Nakagawa et al. 2008 [32]	Brazil	PP	IG: 7 GC: 7	23.6 ± 5.9	M and F 4/10	IG: knee + hip and core CG: knee + mobilisation + stretching	6 weeks 5 days a week (1 supervised)	Pain: VAS Strength: isokinetic dynamometry		PAIN			STRENGTH Isokinetic dynamometry (Nm/kg):		
										Usual VAS: Pre-IG: 3.8 ± 2.1 Post: 1.1 ± 1.2 CG Pre: 4.7 ± 2.6 Post: 4.0 ± 2.6 Maximum VAS: IG Pre: 5.0 ± 2.1 Post: 1.4 ± 1.3 CG Pre: 5.5 ± 1.5 Post: 3.4 ± 1.9		Climbing stairs (VAS): IG Pre: 3.5 ± 3.7 Post: 0.4 ± 0.6 CG Pre: 5.0 ± 3.4 Post: 2.6 ± 2.8 Going down stairs (VAS): IG Pre: 4.5 ± 3.1 Post: 0.3 ± 0.4 CG Pre: 4.7 ± 3.3 Post: 2.0 ± 2.4	KNEES: IG Pre: 264.9 ± 84.8 Post: 318.9 ± 96.8 CG Pre: 283.6 ± 45.0 Post: 301.9 ± 63.4		HIP ABD: IG Pre: 89.1 ± 29.5 Post: 102.2 ± 19.8 CG Pre: 114.6 ± 32.1 Post: 120.4 ± 30.4 RE HIP: IG Pre: 55.5 ± 14.6 Post: 59.4 ± 18.9 CG Pre: 60.4 ± 16.5 Post: 62.9 ± 24.9
Razeghi et al. 2010 [34]	Iran	PP	IG: 16 CG: 16	22.62 ± 2.67	M 0/32	IG: hip + knee CG: knee	4 weeks (Number of days not specified)	Pain: VAS Strength: digital dynamometer (isometric)		PAIN VAS: Pre-test IG: 6.68 ± 1.62 Post: 3.37 ± 1.50 CG Pre: 6.31 ± 1.25 Post: 4.81 ± 1.79			STRENGTH No pre–post values are reported by group - Improvement in strength associated with pain reduction in: Hip flexors: +32.95% Abductors: +34.75% External rotators: +24.21%		
Saad et al. 2018 [39]	Brazil	ITT	IG: 10 CG: 10 SG: 10 CG: 10	IG: 22.5 ± 1.08 CG: 23.2 ± 2.53 SG: 21.3 ± 1.16 CG: 23.2 ± 1.03	M 0/40	IG: hip CG: knee SG: hip and knee stretches CG (NT): no intervention	8 weeks 2 days a week (Supervised)	Pain: VAS Function: AKPS Strength: isometric dynamometry		PAIN VAS: Pre-IG: 5.05 ± 1.27 Post: 0.55 ± 0.8 CG Pre: 6.34 ± 1.85 Post: 0.56 ± 0.89 SG Pre: 4.08 ± 0.82 Post: 0.14 ± 0.25 CG (NT) Pre: 4.03 ± 1.03 Post: 3.69 ± 0.6	FUNCTION AKPS: IG Pre: 77.4 ± 5.46 Post: 91.8 ± 5.67 CG Pre: 76.9 ± 8.72 Post: 90.11 ± 6.11 SG Pre: 79.8 ± 9.21 Post: 91.0 ± 6.62 CG (NT) Pre: 80.8 ± 10.67 Post: 81.9 ± 8.41	STRENGTH Isokinetic dynamometry:			
												HIP ABD: IG Pre: 11.87 ± 2.68 Post: 15.14 ± 2.48 CG Pre: 10.96 ± 2.99 Post: 11.34 ± 3.74 SG Pre: 13.02 ± 6.02 Post: 13.29 ± 5.45 CG (NT) Pre: 12.06 ± 3.58 Post: 11.4 ± 3.22 HIP FLEXION: IG Pre: 14.94 ± 2.99 Post: 16.55 ± 3.53 CG Pre: 11.82 ± 2.82 Post: 13.97 ± 3.55 SG Pre: 14.6 ± 4.95 Post: 15.02 ± 4.49 CG (NT) Pre: 13.42 ± 3.29 Post: 13.42 ± 4.03	HIP ADD: IG Pre: 9.99 ± 2.3 Post: 11.13 ± 1.69 CG Pre: 8.22 ± 3.55 Post: 8.09 ± 2.61 SG Pre: 9.62 ± 4.57 Post: 9.62 ± 4.57 CG (NT) Pre: 8.77 ± 1.68 Post: 8.77 ± 1.68 RE: IG Pre: 7.15 ± 1.21 Post: 8.9 ± 1.07 CG Pre: 7.44 ± 1.52 Post: 7.76 ± 1.87 SG Pre: 8.03 ± 3.49 Post: 8.15 ± 2.86 CG (NT) Pre: 7.72 ± 1.27 Post: 7.09 ± 1.61		RI: IG Pre: 8.88 ± 3.36 Post: 10.99 ± 2.95 CG Pre: 8.48 ± 2.69 Post: 8.59 ± 2.75 SG Pre: 9.61 ± 4.18 Post: 10.4 ± 4.18 CG (NT) Pre: 10.0 ± 2.14 Post: 9.14 ± 1.86 KNEE EXTENSION: IG Pre: 30.38 ± 10.69 Post: 35.23 ± 7.28 CG Pre: 20.86 ± 9.17 Post: 25.26 ± 11.16 SG Pre: 32.07 ± 13.56 Post: 31.85 ± 15.26 CG (NT) Pre: 39.71 ± 9.54 Post: 36.76 ± 10.44
Song et al. 2009 [38]	Taiwan	ITT	IG: 29 CG: 30 CG (NT): 30	IG: 38.6 ± 10.8 CG: 40.2 ± 9.9 CG: 43.9 ± 9.8	H and M 20/69	IG: hip + knee CG: knee CG (NT): no intervention	8 weeks 3 days a week	Pain: VAS (worst pain in the past week) Function: Lysholm scale		PAIN VAS: Pre-intervention mean: 4.80 ± 2.26 Post: 2.62 ± 2.51 CG Pre: 4.85 ± 2.49 Post: 2.26 ± 2.20 CG (NT) Pre: 4.99 ± 2.18 Post: 4.81 ± 2.55			FUNCTION Lysholm: IG Pre: 74.8 ± 12.1 Post: 85.7 ± 8.5 CG Pre: 75.7 ± 12.8 Post: 86.5 ± 10.4 CG (NT) Pre: 75.1 ± 9.3 Post: 75.7 ± 10.9		
Yue et al. 2025 [24]	China	ITT	IG: 9 CG 9	IG: 24.90 ± 5.30 CG (NT): 25.67 ± 5.85	H 18/0	IG: hip CG (NT): no intervention	6 weeks 3 days a week	Pain: VAS Strength/performance: explosive power (EP)		PAIN VAS: Pre-test GI: 4.76 ± 1.17 Post: 2.88 ± 1.29 CG (NT) Pre: 4.84 ± 1.11 Post: 4.59 ± 1.18			Strength/performance (EP): IG Pre: 2840.04 ± 214.59 Post: 3087.32 ± 175.32 CG (NT) Pre: 2827.26 ± 214.29 Post: 2692.48 ± 167.16		
Sahin et al. 2016 [35]	Turkey	PP	IG: 25 CG: 25	IG: 33.3 ± 6.5 CG: 35.0 ± 5.9	M 0/50	IG: hip + knee CG: knee	6 weeks 5 days a week (under supervision) + 6 additional sessions at home.	Pain: VAS Function: Kujala score and functional tests (step-down, one-leg squat, hop) Strength: isokinetic dynamometry (Biodex)		PAIN VAS: Changes reported (no exact pre–post averages): - Both groups show improvement - The IG shows significantly greater improvement than the CG in: resting, walking, running, climbing stairs and squatting.		FUNCTION Kujala: approx. IG Pre: ~71.4 Post (12W): 83.0 ± 6.8 CG Pre: ~72.4 Post (12W): 77.9 ± 6.6 Objective function (tests): One-leg squat → better in the IG Step-down → better in the IG Hop test → no clear differences.	STRENGTH Isokinetic dynamometry: Hip abduction (60°/s): Greater improvement in the IG vs. the CG (p = 0.004) - External rotation (60°/s): Greater improvement in the IG		

## References

[1] Alammari A., Spence N., Narayan A., Karnad S.D., Ottayil Z.C. (2023). Effect of hip abductor and lateral rotator muscle strengthening on pain and functional outcome in adult patients with patellofemoral pain: A systematic review and meta-analysis. J. Back Musculoskelet. Rehabil..

[2] Crossley K.M., Stefanik J.J., Selfe J., Collins N.J., Davis I.S., Powers C.M., McConnell J., Vicenzino B., Bazett-Jones D.M., Esculier J.-F. (2016). 2016 Patellofemoral pain consensus statement from the 4th International Patellofemoral Pain Research Retreat, Manchester. Part 1: Terminology, definitions, clinical examination, natural history, patellofemoral osteoarthritis and patient-reported outcome measures. Br. J. Sports Med..

[3] Smith B.E., Selfe J., Thacker D., Hendrick P., Bateman M., Moffatt F., Rathleff M.S., Smith T.O., Logan P. (2018). Incidence and prevalence of patellofemoral pain: A systematic review and meta-analysis. PLoS ONE.

[4] Nejati P., Forogh B., Moeineddin R., Baradaran H.R., Nejati M. (2011). Patellofemoral pain syndrome in Iranian female athletes. Acta Med. Iran..

[5] Dolak K.L., Silkman C., McKeon J.M., Hosey R.G., Lattermann C., Uhl T.L. (2011). Hip strengthening prior to functional exercises reduces pain sooner than quadriceps strengthening in women with patellofemoral pain syndrome: A randomised clinical trial. J. Orthop. Sports Phys. Ther..

[6] Zare Bidoki F., Haj Lotfalian M. (2026). The effects of combined balance and perturbation training on gluteal muscle activity and hip kinematics in women with patellofemoral pain syndrome: A randomised controlled trial. BMC Musculoskelet. Disord..

[7] Drake R.L., Vogl W., Mitchell A.W., Tibbitts R., Richardson P. (2023). Gray’s Basic Anatomy.

[8] Wheatley M.G.A., Rainbow M.J., Clouthier A.L. (2020). Patellofemoral Mechanics: A Review of Pathomechanics and Research Approaches. Curr. Rev. Musculoskelet. Med..

[9] Zaffagnini S., Dejour D., Grassi A., Bonanzinga T., Marcheggiani Muccioli G.M., Colle F., Raggi F., Benzi A., Marcacci M. (2013). Patellofemoral anatomy and biomechanics: Current concepts. Joints.

[10] Senavongse W., Amis A.A. (2005). The effects of articular, retinacular, or muscular deficiencies on patellofemoral joint stability: A biomechanical in vitro study. J. Bone Jt. Surg. Br..

[11] Yamada Y., Toritsuka Y., Horibe S., Sugamoto K., Yoshikawa H., Shino K. (2007). In vivo movement analysis of the patella using a three-dimensional computer model. J. Bone Jt. Surg. Br..

[12] Dixit S., Difiori J.P., Burton M., Mines B. (2007). Management of patellofemoral pain syndrome. Am. Fam. Physician.

[13] Rogan S., Haehni M., Luijckx E., Dealer J., Reuteler S., Taeymans J. (2019). Effects of hip abductor muscle exercises on pain and function in patients with patellofemoral pain: A systematic review and meta-analysis. J. Strength Cond. Res..

[14] Halabi M.H., Alturkistani B.A., Abuhadi R.H., Garout A.N., Almuqbil F.B., Alshehri M.S. (2025). The Efficacy of Hip and Knee Muscle Strengthening Versus Knee Muscle Strengthening Alone in Managing Patellofemoral Pain Syndrome: A Systematic Review and Meta-Analysis. Musculoskelet. Care.

[15] Fukuda T.Y., Melo W.P., Zaffalon B.M., Rossetto F.M., Magalhães E., Bryk F.F., Martin R.L. (2012). Hip posterolateral musculature strengthening in sedentary women with patellofemoral pain syndrome: A randomized controlled clinical trial with 1-year follow-up. J. Orthop. Sports Phys. Ther..

[16] Fukuda T.Y., Rossetto F.M., Magalhães E., Bryk F.F., Lucareli P.R.G., De Almeida Carvalho N.A. (2010). Short-term effects of strengthening the hip abductors and lateral rotators in women with patellofemoral pain syndrome: A randomised controlled clinical trial. J. Orthop. Sports Phys. Ther..

[17] Baldon R de M., Serrao F.V., Silva R.S., Piva S.R. (2014). Effects of functional stabilisation training on pain, function, and lower extremity biomechanics in women with patellofemoral pain: A randomised clinical trial. J. Orthop. Sports Phys. Ther..

[18] De Oliveira Silva D., Briani R.V., Pazzinatto M.F., Ferrari D., Aragão F.A., De Albuquerque C.E., Alves N., de Azevedo F.M. (2015). Reliability and differentiation capability of dynamic and static kinematic measurements of rearfoot eversion in patellofemoral pain. Clin. Biomech..

[19] Gragnani B.C., Hart H.F., Forsyth A., Barton C.J., De Oliveira Silva D. (2026). Patellofemoral pain is a multifactorial complex condition; are we missing a multidisciplinary approach to its management? Time for a paradigm shift. Open Access J. Sports Med..

[20] Lun V., Meeuwisse W.H., Stergiou P., Stefanyshyn D. (2004). Relationship between running injuries and static lower limb alignment in recreational runners. Br. J. Sports Med..

[21] Lack S., Neal B., De Oliveira Silva D., Barton C. (2018). How to manage patellofemoral pain—Understanding the multifactorial nature and treatment options. Phys. Ther. Sport.

[22] Khayambashi K., Mohammadkhani Z., Ghaznavi K., Lyle M.A., Powers C.M. (2012). The effects of isolated hip abductor and external rotator muscle strengthening on pain, health status, and hip strength in women with patellofemoral pain: A randomised controlled trial. J. Orthop. Sports Phys. Ther..

[23] Ireland M.L., Willson J.D., Ballantyne B.T., Davis I.M.C. (2003). Hip strength in women with and without patellofemoral pain. J. Orthop. Sports Phys. Ther..

[24] Yue W., Shuang R., Hongshi H., Yingfang A., Bo G. (2025). A Study on the Effects of Gluteal Muscle Activation on the Electromyography of Lower Limb Muscles in Young Male Patients with Patellofemoral Pain Syndrome. Orthop. Surg..

[25] Teichtahl A.J., Wulidasari E., Brady S.R.E., Wang Y., Wluka A.E., Ding C., Giles G.G., Cicuttini F.M. (2015). A large infrapatellar fat pad protects against knee pain and lateral tibial cartilage volume loss. Arthritis Res. Ther..

[26] Moher D., Liberati A., Tetzlaff J., Altman D.G. (2009). Preferred reporting items for systematic reviews and meta-analyses: The PRISMA statement. BMJ.

[27] Higgins J.P.T., Thomas J., Chandler J., Cumpston M., Li T., Page M.J., Welch V.A. (2019). Cochrane Handbook for Systematic Reviews of Interventions.

[28] Page M.J., McKenzie J.E., Bossuyt P.M., Boutron I., Hoffmann T.C., Mulrow C.D., Shamseer L., Tetzlaff J.M., Akl E.A., Brennan S.E. (2021). The PRISMA 2020 statement: An updated guideline for reporting systematic reviews. BMJ.

[29] Sherrington C., Herbert R.D., Maher C.G., Moseley A.M. (2000). PEDro. A database of randomised trials and systematic reviews in physiotherapy. Man. Ther..

[30] Sterne J.A.C., Savović J., Page M.J., Elbers R.G., Blencowe N.S., Boutron I., Cates C.J., Cheng H.Y., Corbett M.S., Eldridge S.M. (2019). RoB 2: A revised tool for assessing risk of bias in randomised trials. BMJ.

[31] Guyatt G.H., Oxman A.D., Vist G.E., Kunz R., Falck-Ytter Y., Alonso-Coello P., Schünemann H.J. (2008). GRADE: An emerging consensus on rating quality of evidence and strength of recommendations. BMJ.

[32] Nakagawa T.H., Muniz T.B., Baldon R de M., Dias Maciel C., de Menezes Reiff R.B., Serrão F.V. (2008). The effect of additional strengthening of hip abductor and lateral rotator muscles in patellofemoral pain syndrome: A randomised controlled pilot study. Clin. Rehabil..

[33] Ismail M.M., Gamaleldein M.H., Hassa K.A. (2013). Closed kinetic chain exercises with or without additional hip strengthening exercises in the management of patellofemoral pain syndrome: A randomised controlled trial. Eur. J. Phys. Rehabil. Med..

[34] Razeghi M., Etemadi Y., Taghizadeh S., Ghaem H. (2010). Could hip and knee muscle strengthening alter the pain intensity in patellofemoral pain syndrome?. Iran. Red. Crescent Med. J..

[35] Şahin M., Ayhan F.F., Borman P., Atasoy H. (2016). The effect of hip and knee exercises on pain, function, and strength in patients with patellofemoral pain syndrome: A randomised controlled trial. Turk. J. Med. Sci..

[36] Crossley K.M., Bennell K.L., Cowan S.M., Green S. (2004). Analysis of outcome measures for people with patellofemoral pain: Which are reliable and valid?. Arch. Phys. Med. Rehabil..

[37] Ferber R., Bolgla L., Earl-Boehm J.E., Emery C., Hamstra-Wright K. (2015). Strengthening of the hip and core versus knee muscles for the treatment of patellofemoral pain: A multicentre randomised controlled trial. J. Athl. Train..

[38] Song C.Y., Lin Y.F., Wei T.C., Lin D.H., Yen T.Y., Jan M.H. (2009). The added benefit of hip adduction in leg-press exercises for patients with patellofemoral pain syndrome: A randomised controlled trial. Phys. Ther..

[39] Saad M.C., de Vasconcelos R.A., de Oliveira Mancinelli L.V., de Barros Munno M.S., Liporaci R.F., Grossi D.B. (2018). Is hip strengthening the best treatment option for women with patellofemoral pain? A randomised controlled trial of three different types of exercises. Braz. J. Phys. Ther..

[40] Hansen R., Brushøj C., Rathleff M.S., Magnusson S.P., Henriksen M. (2023). Quadriceps or hip exercises for patellofemoral pain? A randomised controlled equivalence trial. Br. J. Sports Med..

[41] Hott A., Brox J.I., Pripp A.H., Juel N.G., Paulsen G., Liavaag S. (2019). Effectiveness of isolated hip exercise, knee exercise, or free physical activity for patellofemoral pain: A randomised controlled trial. Am. J. Sports Med..

[42] Hart L. (2010). Supervised exercise versus usual care for patellofemoral pain syndrome. Clin. J. Sport Med..

[43] Powers C.M. (2010). The influence of abnormal hip mechanics on knee injury: A biomechanical perspective. J. Orthop. Sports Phys. Ther..

[44] Maher C.G., Sherrington C., Herbert R.D., Moseley A.M., Elkins M. (2003). Reliability of the PEDro Scale for Rating Quality of Randomised Controlled Trials. Phys. Ther..

[45] Cano-Uceda A., García-Fernández P., Peuyadé-Rueda B., Cañuelo-Marquez A.M., Solís-Mencía C., Lucio-Allende C., De Sousa-De Sousa L., Maté-Muñoz J.L. (2025). From Evidence to Practice: A Systematic Review and Meta-Analysis on the Effects of Supervised Exercise on Fatigue in Breast and Prostate Cancer Survivors. Appl. Sci..

[46] Schulz K.F., Altman D.G., Moher D. (2010). CONSORT 2010 statement: Updated guidelines for reporting parallel-group randomised trials. Ann. Intern Med..

[47] Cano-Uceda A., De Sousa-De Sousa L., Bueno-Fermoso R., Rozalén-Bustín M., Lucio-Allende C., Barba-Ruiz M., Sánchez-Barroso L., Maté-Muñoz J.L., García-Fernández P. (2025). Improving Quality of Life Through Supervised Exercise in Oncology: A Systematic Review and Meta-Analysis of Randomised Trials in Breast and Prostate Cancer. J. Funct. Morphol. Kinesiol..

[48] Lu Z., Li X., Sun D., Song Y., Fekete G., Kovács A., Gao Z., Zheng J., Xiang L., Gu Y. (2025). Computationally tuned dual-layer lattice pads adapted to gait-induced pressure distribution. npj Adv. Manuf..

[49] Lu Z., Li X., Sun D., Song Y., Fekete G., Kovács A., András K., Gu Y. (2025). Parametric cushioning lattice insole based on finite element method and machine learning: A preliminary computational analysis. J. Biomech..

